# Short-Term Demand Forecasting of Urban Online Car-Hailing Based on the K-Nearest Neighbor Model

**DOI:** 10.3390/s22239456

**Published:** 2022-12-03

**Authors:** Yun Xiao, Wei Kong, Zijun Liang

**Affiliations:** School of Urban Construction and Transportation, Hefei University, Hefei 230606, China

**Keywords:** traffic engineering, urban online car-hailing, short-term forecasting, K-nearest neighbor

## Abstract

Accurately forecasting the demand of urban online car-hailing is of great significance to improving operation efficiency, reducing traffic congestion and energy consumption. This paper takes 265-day order data from the Hefei urban online car-hailing platform from 2019 to 2021 as an example, and divides each day into 48 time units (30 min per unit) to form a data set. Taking the minimum average absolute error as the optimization objective, the historical data sets are classified, and the values of the state vector T and the parameter K of the K-nearest neighbor model are optimized, which solves the problem of prediction error caused by fixed values of T or K in traditional model. The conclusion shows that the forecasting accuracy of the K-nearest neighbor model can reach 93.62%, which is much higher than the exponential smoothing model (81.65%), KNN1 model (84.02%) and is similar to LSTM model (91.04%), meaning that it can adapt to the urban online car-hailing system and be valuable in terms of its potential application.

## 1. Introduction

An important part of the urban transportation system, online car-hailing has become the transportation choice for more and more urban residents. In 2020, there were 214 online car-hailing platforms across China, with an average 21 million daily orders taking place. Accurately forecasting the travel demand for online car-hailing is of great significance for regard to reducing vehicle idling, improving operational efficiency, and reducing traffic congestion and energy consumption [1,2,3,4]. Reasonable forecasting results can provide data support for vehicle scheduling and allocation, which is beneficial for solving problems caused by asymmetric supply and demand, as well as maximizing benefits for passengers, drivers, and ride-hailing platforms [5].

In the early stages in the development of online car-hailing, many scholars used questionnaires or interviews to make judgments concerning the future development status and changing trends with regard to the scale of travel. However, the survey process comes with problems such as low efficiency, not being able to guarantee timeliness, and in particular, a lack of an accurate description of travel demand [6]. With the accumulation of historical data on online car-hailing, scholars have carried out quantitative forecasting research on the operation status of online car-hailing. The main representative models are the time series model [7], historical average model, Kalman filter model, and the linear regression analysis method [8]. In recent years, intelligent forecasting models have gradually become used widely in urban traffic prediction, and mainly comprise neural networks, non-parametric regression prediction methods, support vector machines, and other methods [7]. Long short-term memory network (LSTM) is a time recurrent neural network, which is suitable for processing and predicting important events with relatively long intervals and delays in time series, and is suitable for traffic flow modeling and prediction. The unique unit structure of LSTM provides great advantages in dealing with temporal short-term traffic flow prediction problems. Ma et al. (2015) constructed a prediction model based on LSTM which effectively captured nonlinear traffic dynamics and automatically determined the optimal time delay. The efficiency of the model was verified by measured data [9]. Tian et al. (2018) used LSTM to predict traffic flow in the absence of data, and achieved higher accuracy than traditional methods [10]. Ye et al. (2020) combined deep learning model of LSTM + Attention for short-term demand forecasting of online car-hailing. The results show that the LSTM + Attention model is superior to other models [1]. Niu K et al. (2019) proposed a new region partition-aided long short-term memory neural network for car-hailing service demand prediction [11]. In 2017, the Transformer structure composed entirely of attentional mechanisms was proposed. In recent years, there have also been some studies applying it to traffic flow prediction [12]. Li (2021) constructed a lightweight traffic flow prediction model based on Transformer, which can predict the traffic volume of any area in the next period [13]. In order to predict the travel demand of online car-hailing more accurately, Bi et al. (2022) proposed a new spatio-temporal prediction method model based on Transformer architecture. Compared with the three most commonly used models, the results show that the model has the best prediction accuracy and prediction accuracy training speed [14]. With the rapid development of online big data technology and the rise of non-parametric regression models in the field of forecasting, forecasting methods represented by the K-nearest neighbor method have started to be introduced into the field of transportation. The main technical ideas of this method include predictions being performed by searching for the K records in the historical database that are most similar to the feature vector of the predicted value, finding the K historical state vectors that are most similar to the current state vector, and, after weighting the historical state vector, the current state value being predicted. Smith et al. (1997) used the same historical data set and used the time series model, the ARIMA model, and the neural network model to forecast, and found that the K-nearest neighbor method had the best stability and error accuracy [15]. There are three core problems in the K-nearest neighbor method: The first concern involves the issue of identification and classification of different patterns from historical data when classifying the historical data set. The second concern involves constructing a state vector: in the field of transportation, historical traffic data are often divided into several samples, each of which is a state vector. The third problem involves determining the K-value algorithm.

Regarding the classification of historical data sets, Liu Z et al. (2017) took the historical intersection traffic volume data set on the Portland Expressway in the United States as a whole, and did not classify them according to time or space distribution [16]. Lin P et al. (2018) divided the passenger flow at the Guangzhou South Railway Station into ten daily passenger flow development models, but still used the whole-day historical data set for prediction [17]. Wang X et al. (2015) analyzed the travel time of the Shanghai–Nanjing Expressway from Shanghai to Nanjing; according to changes in the expressway traffic, the historical data set was divided into a morning peak, an evening peak, a flat peak, and other categories, and a travel time prediction was carried out separately. However, the classification of historical data was mainly based on subjective judgments of traffic wave-forms, and there was a lack of scientific verification or analysis of data classification [18].

The state vector is used to describe the comparison standard at different times. Since the traffic volume or passenger volume has the autocorrelation of time series, many scholars choose T times before the predicted time as the feature vector. For the selection of T, most scholars usually divide it according to one day or one week. For example, to predict the traffic volume of a certain hour, the preferred unit of time is days, and the first 23 h are used as the state vector. To predict the traffic data on a certain day, the week is used as the unit, and the first 6 days are used as the state vector [19]. This method can lead to large errors in some fields occurring. Some scholars have noticed that the selection of T has a significant impact on prediction accuracy and the T value is selected by the correlation coefficient, but in-depth research on the relationship between the correlation coefficient ρ and the T value is lacking [18]. Porwik P et al. found that “nearest neighbors” always appeared in a relatively narrow time period and that T was below 10 time periods in most cases, but did not propose a specific calculation method for the T value. By traversing the possible T values, Wang X et al. (2019) proposed a method for selecting the T value based on the smallest prediction error, which has great reference significance [20].

In the K-nearest neighbor prediction algorithm, the value of K affects the accuracy of any short-term traffic flow prediction; choosing an appropriate K value plays a crucial role in short-term traffic flow prediction. There are many research theories on the selection of a K value. Zhou X et al. (2006) believe that the randomness of traffic flow is too high, and propose setting the number of K to different values according to different modes, thereby optimizing the prediction accuracy [21]. Yu B et al. (2012) believe that after the K value satisfies the expected value, a smaller K value can be taken to improve the model calculation speed [22]. Zhu B (2019) used a K-means clustering algorithm to divide the passenger flow into data sets under different conditions, and then performed K-Fold cross-validation on the data sets under different conditions to determine the value of the number of neighbors K under different conditions [23]. Lei S (2017) took 20 sets of data from the historical database according to the three traffic states for experimental verification; a value of K from 5 to 12 was taken in order to carry out short-term traffic flow predictions, and they were compared so as to obtain the prediction results on short-term traffic flow. Compared with the constant K value, the prediction method using the variable K value has better prediction accuracy [24]. The above scholars have proposed different approaches to K-value calculation from different perspectives, but their main core remains basically the same, namely the setting a range of K-value values and determining the evaluation method of prediction accuracy, traversing all K-values and determining the optimal K-value with the goal of the highest prediction accuracy or prediction accuracy reaching a certain threshold.

Existing research provides good methods for the short-term forecasting of urban online car-hailing demand, but there are also some shortcomings. Some model methods, such as the time series method, which perform well in the field of long-term forecasting are not fully adapted to the characteristics of a large quantity of data, strong volatility, and strong timeliness in the operation of online car-hailing, and expose a large deficiency in the aspect of prediction delay [18]. The K-nearest neighbor model can better adapt to these features. The algorithm is simple and the theory is mature, which can be used for classification and regression, and it is more suitable for automatic classification of class domains with large sample size. However, there is still room for optimization in the selection of historical data set classification, the state vector K, and the K value in the existing K-nearest neighbor methods. Therefore, based on the order data of urban online car-hailing platform, aiming at the characteristics of online car-hailing operation, this paper explores the optimization of K and T values to improve the K-nearest neighbor algorithm model. In response to these problems, this paper aims to forecast the demand for online car-hailing. First, the data set is divided into “*n*” categories according to the unit of day; secondly, the state vector dimension T takes values from 1 to *n* − 1 and calculates the prediction error under different values. At the same time, for each K, the possible value range of K is traversed, and then the T and K are found with the highest prediction accuracy. Finally, the errors from different forecasting methods are compared in order to verify the scientific nature and feasibility of the research method.

## 2. Methods

### 2.1. Basic Idea of the K-Nearest Neighbor Algorithm

The K-nearest Neighbor (KNN) algorithm is an efficient non-parametric classification algorithm proposed by Cover and Hart (1967) [25]. It makes predictions by searching for the K records in a historical database that are most similar to the feature vector of the predicted value. It has strong stability and has been widely used in classification, regression, and pattern recognition in recent years. Based on the improved K-nearest neighbor model algorithm, this paper establishes a short-term forecasting model for online car-hailing demand, and constructs the basic flow of the algorithm for the short-term prediction of urban online car-hailing demand as follows:

(1) The original database is cleaned of historical orders, and one day is divided into 48 units (30 min per unit), which builds an order data set;
(2) The search mechanism of the model is determined, which is composed of the state vector, the distance measurement method, the value of the state vector T and the number of neighbors K;
(3) The K nearest neighbor prediction algorithm is determined and the prediction result is calculated;
(4) The mean absolute percentage error (*MAPE*) is used as an indicator in order to evaluate the prediction results, and a comparative analysis is conducted.

**Figure 1:**
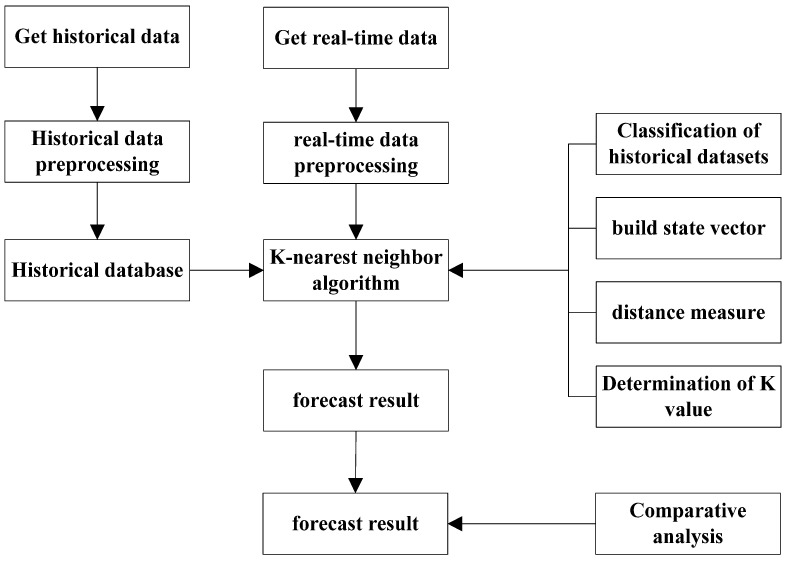
Basic flow chart of the K-nearest neighbor algorithm.

The algorithm flow chart is shown in Figure 1.

### 2.2. Classification of Historical Data

**Figure 2:**
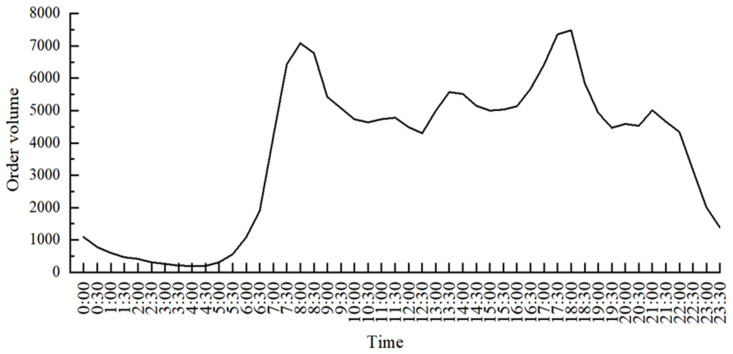
Changes in the average daily order volume of online car hailing.

This paper divides 24 h a day into 48 time units, each time unit is 30 min, counts the number of online car-hailing orders in each time unit from the platform, and analyzes its change trends. In general, prediction accuracy increases as the classification of the data set increases, so the data is divided into 48 categories in order to improve the prediction accuracy, that is, 1 (00:00), 2 (00:30), 3 (01:00), 4 (01:30)...48 (23:30), as shown in Figure 2.

### 2.3. Constructing the State Vector

The state vector is the standard for comparing the current data with the historical data. Generally, the factors that are most relevant to the prediction object are selected to predict [22]. The real-time data on the forecast day can fully reflect the change trend of its passenger flow. Therefore, its nearest neighbor can be found in the historical database through variation of passenger flow presented by the real-time data, and the passenger flow at the next time can be calculated through the change law of real-time data and historical data so as to construct the state vector.

(1) $$D_{n}=(x_{n1},x_{n2},\ldots \ldots ,x_{n(T-1)})$$

In the formula, *n* represents the *n*th day, and when *n* is 0, it represents the forecast day; *x_n_*_(_*_T −_*_1)_ is the number of urban online car-hailing orders in the period *T* − 1 on the *n*th day before the forecast date; because T must be smaller than the dimension of the data set (48), the value range of T is [1, 47]. In order to obtain the optimal T value, this paper intends to traverse all T values with the highest prediction accuracy as the goal.

### 2.4. Distance Measurement Method

The distance measurement method is used to measure the approximation of each historical sample in the historical database and the current data. Many previous studies have chosen Euclidean distance as the distance measurement method [16,17,18]. And Euclidean distance is a time series alignment method aligned according to time points, calculate the sum of Euclidean distances between the same time points as the distance between two time series, which is suitable for prediction on online car-hailing demand comparison at different time points.

(2) $$d_{n}=\sqrt{\sum_{i=1}^{T-1}{\left(x_{n}{}_{i}-x_{0j}\right)}^{2}}$$

In the formula, *d_n_* is the distance between the data from each period of the forecast day and the data from each period of the historical day, *x_ni_* is the number of online car-hailing orders in the city in the *i*th period of the nth day before the forecast date, and *x*_0*j*_ is the delivery order volume for the city in the *j*th period of the forecast day.

### 2.5. Evaluation Method

Commonly used evaluation model indicators are Mean Absolute Error (*MAE*), Mean Squared Error (*MSE*), Mean Absolute Percentage Error (*MAPE*), and Mean Squared Percentage Error (*MSPE*). *MAPE* is standardized on the basis of the other evaluation indicators, which more intuitively reflects the prediction accuracy and difference of this model and has good adaptability. Therefore, this paper uses the mean absolute percentage error (*MAPE*) index as the performance evaluation of the model. The smaller the *MAPE*, the better the model is. Its calculation formula is as follows:

(3) $$MAPE(T_{i},K_{j},D_{e})=\frac{1}{n}\sum_{i=1}^{n}MAPE(T_{i},K_{j},D_{e})$$

In the formula, *n* is the number of samples, *x_i_* is the actual value of the sample, and ${\hat{x}}_{i}$ is the predicted value of the sample.

### 2.6. Prediction Algorithms

The prediction algorithm is used to describe a way to use the searched K groups of neighbors to predict the demand at the next moment:

(4) $${\hat{x}}_{o}=\sum_{i=1}^{K}\frac{d_{i}^{-1}}{\sum_{i=1}^{K}d_{i}^{-1}}x_{i}$$

In the formula, ${\hat{x}}_{0}$ is the predicted value for the current data, *x_i_* is the order quantity corresponding to the *i*th neighbor searched in the historical database, and *d_i_* is the distance between the current data and the *i*th neighbor.

### 2.7. Calibration of Adaptive K Value and T Value

The way in which a reasonable K value and T value are chosen is key to the prediction model [22]. In this paper, the prediction scenarios are classified, the error rate of change of the prediction results under different K and T values is calculated for each category, and the prediction accuracy is compared to select the better value.

Step 1: The predicted scenarios are classified, and the predicted values are divided into N categories, set *n* = *n_i_*.

Step 2: The value of the state vector T in the scene is determined, and T = *T_i_*, *T_i_* ∈ [1, 47] is set.

Step 3: K = *K_j_*, *K_j_* ∈ [1, K*_max_*] is set, and K*_max_* is comprehensively determined according to the quantity of historical data.

Step 4: Any one-day *D_e_* from the historical data set is selected as the test data set, and other *n* − 1 days are selected as the training data set, in which each data set has 48 pieces of data. Forty-eight and a half hours of urban online car-hailing orders are represented.

Step 5: The mean absolute error percentage is calculated for the test data set De under *T_i_* and *K_j_*.

(5) $$MAPE(T_{i},K_{j},D_{e})=\frac{1}{48}\sum_{i=1}^{48}\left|\frac{\overset{\wedge }{X_{i}}-X_{i}}{X_{i}}\right|$$

Step 6: The mean absolute error percentage is calculated for all test data sets *D_e_* under *T_i_* and *K_j_*.

(6) $$MAPE(T_{i},K_{j},D_{e})=\frac{1}{n}\sum_{i=1}^{n}MAPE(T_{i},K_{j},D_{e})$$

Step 7: When the minimum value of *MAPE* is obtained, the predicted corresponding *T_i_* and *K_j_* are the optimal values of the data set in the *n_i_* prediction scenario.

## 3. Empirical Analysis

### 3.1. Research Data

The data originate from the online car-hailing supervision platform. The research area is Hefei City, which is the capital of the Anhui Province. It is a mega city with a permanent population of 8.087 million. In October 2018, 19 online car-hailing platforms in Hefei City obtained online car-hailing business licenses, and about 300,000 vehicles were registered. There are about 15,000 active vehicles, and about 200,000 orders are completed every day for online car-hailing in Hefei [26]. The data collection period is 265 days from 1 August 2019 to 4 February 2021. One piece of order data is generated every 30 min, and 48 pieces of order data are generated in one day. The original data include three fields: the first column is “date”, the second column is “time”, and the third column is “order volume”. The original data are shown in Table 1 below.

The raw data do not meet the conditions for direct analysis, and must be processed to be converted into standardized data. First, the null values, abnormal values, and so on, are deleted in the original order data; secondly, considering that the data during the novel COVID-19 epidemic in early 2020 were quite different, the order outliers during the epidemic were removed; finally, the string with time information was converted into a timestamp in order to adjust the data format, and the data scattered in different columns and different rows were integrated to make it a whole set of data for each day. Each group of data has one piece of data for every 30 min within 24 h of a day, forming 48 pieces of order volume data, with a total of 265 days of data, some of which are shown in Table 2 below.

### 3.2. Constructing the State Vector

The reasonable construction of the state vector is an important factor that affects prediction accuracy [27]. In contrast to some scholars sets the data dimension T of the state vector as a fixed value, the paper adopts the changing T of the state vector, which takes values from 1~*n* − 1. The prediction of the 23:30 data entry is taken as an example, and the state vector T at 23:30 can be [1–47]; that is, the state vector of 23:30 according to the value of T is (T47), (T47, T46), (T47, T46, T45)……(T47, T46, T45, T44,……T3, T2, T1). If the data entry at 23:00 is predicted, the state vector according to the value of T is (T46), (T46, T45), (T46, T45, T44)……(T46, T45, T44, ……T3, T2, T1), (T46, T45, T44,……T3, T2, T1, T48). *T_i_* represents the *i*th period, and so on.

### 3.3. Selection of K Value and T Value

If the K value is too large or too small, it is not conducive to improving prediction accuracy. If the K*_max_* (Maximum value of K parameter) value is too large in the algorithm traversal process, the calculation time will be too long and the efficiency will be too low. The value of K*_max_* is generally 20%–40% of the overall data sample [17,18,19], and this paper determines K*_max_* = 30.

When T = 1, the state vector has only one value, and the number of search data sets is 1. Based on the standardized 265-day data, the adaptive K-value algorithm is used. Starting from the first day, the first day is used as the test set, and the remaining 264 days are used as the training set. The corresponding *MAPE* from K = 1 to K = 30 in the test set on the first day is calculated every 30 min. Then, the next day is used as the test set, and the remaining 263 days are used as the training set. Similarly, the corresponding *MAPE* from K = 1 to K = 30 per day is calculated. The above steps are repeated until all 265 days of data are traversed. From Formula (3), the prediction accuracy of all data in the 265 days under the condition of T = 1 is obtained, and the average prediction accuracy of the 48-time unit per day in the 265 days is calculated separately.

When T = 2, the state vector has 2 values, and the number of search data sets is 2. Based on the standardized 265-day data, the adaptive K-value algorithm is used, and the above steps are repeated to obtain the prediction accuracy of all data in 265 days in the case of T = 2. Furthermore, the average prediction accuracy of the 48-time unit per day in the 265 days was calculated separately.

For analysis, the corresponding average prediction accuracy of each day is obtained from T = 3 to T = 47, and some data are shown in Table 3.

**Figure 3:**
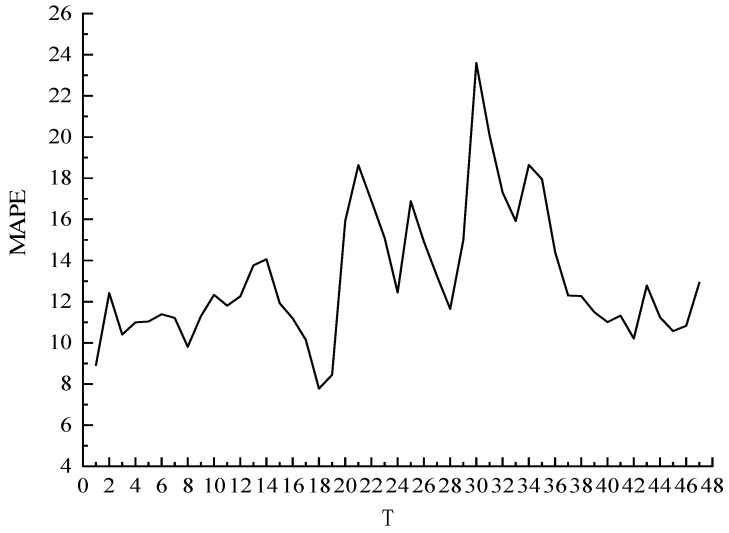
Average *MAPE* values at different T values.

The average absolute percentage error (*MAPE*) of the prediction results for different T values is obtained by averaging the 48 time period *MAPE* values corresponding to the 47 T values of T [1, 47], as shown in Figure 3 below.

**Figure 4:**
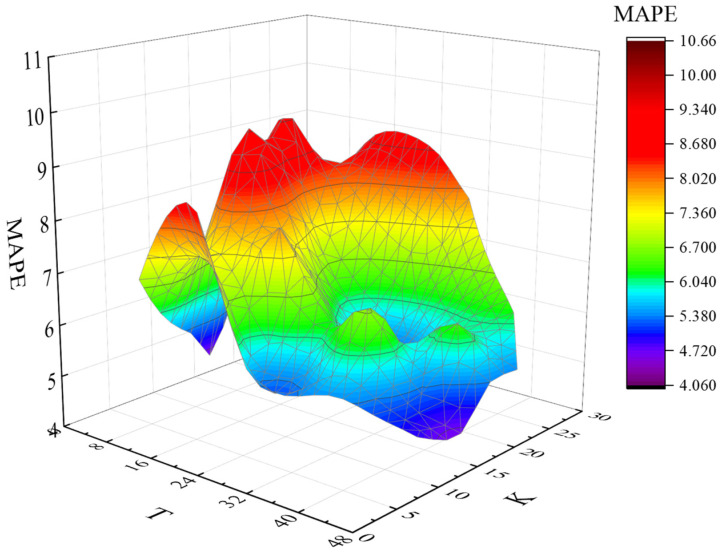
The optimal T value, K value and *MAPE* (00:00).

It can be seen from Figure 3 that the average error gap under different T values is large, and the overall *MAPE* value fluctuates between 7.7% and 17.3%. Taking the prediction data at 00:00 as an example, the optimal T value and the optimal K value were obtained with the highest prediction accuracy (minimum *MAPE*) as the goal. The prediction results are shown in Figure 4. The color depth in the figure represents the prediction accuracy, and the deeper the color, the more accurate the prediction. The optimal T value and K value corresponding to category 1 are T = 3 and K = 15, and the prediction accuracy is up to 96%.

**Figure 5:**
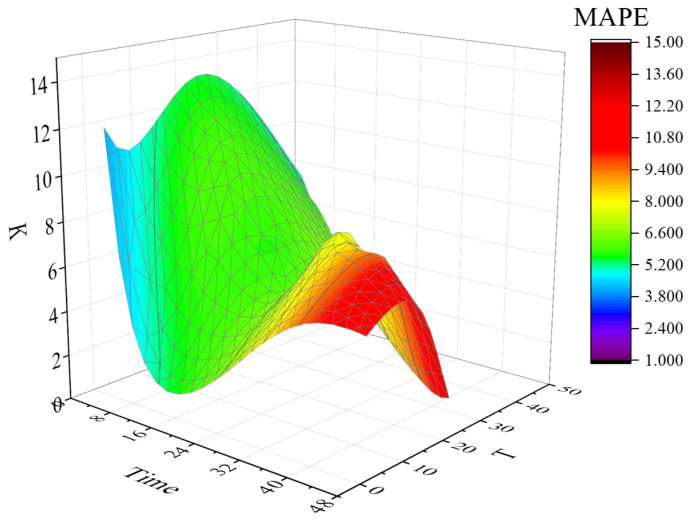
The corresponding relationship between T value, K value and *MAPE* value at different times.

The other category prediction methods are the same as above, and the optimal T value, K value and *MAPE* value corresponding to 48 categories are obtained by analogy, the minimum average absolute error percentage of all kinds of data (1–48) is less than 12%. Among them, the prediction accuracy of the third type is the highest, and the prediction error is the smallest (4.04%). The corresponding optimal T value is 3, and the K value is 3. The prediction accuracy of category 48 is the lowest, and the prediction error is the largest (11.18%). The corresponding optimal T value is 1, and the K value is 6. Overall, the prediction accuracy is better when T value is 18 and K value is [4,8], and the whole day prediction accuracy is 93% as shown in Figure 5.

## 4. Discussion

**Figure 6:**
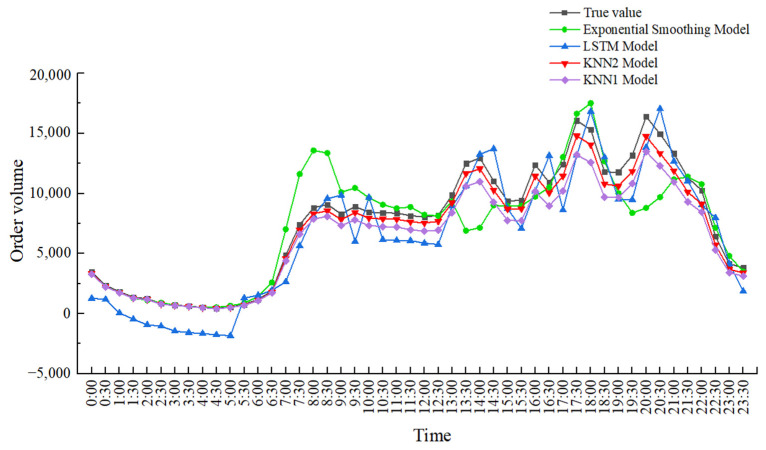
Comparison of the predicted values of online car-hailing demand by different models.

According to the characteristics of online car-hailing order data, a method of optimizing K value and T value to improve the K-nearest neighbor algorithm model is proposed. To better illustrate the prediction effect, the exponential smoothing prediction model, KNN1 model, LSTM model and KNN2 model are used to predict the online car-hailing order volume on 31 January 2021 and compare the results, as shown in Figure 6. In addition, this paper selects the period 14:30 on 31 January 2021 as the prediction analysis sample. 

The exponential smoothing model, also known as exponential smoothing, is an important time series forecasting method. This paper uses SPSS for the exponential smoothing forecast, and the operation process is as follows: first, the date format is defined as a day; second, a time series prediction model is created, an exponential smoothing model is selected, and the steps are followed to complete the settings in order to obtain the prediction result value. This predicted value and the *MAPE* calculation formula are used to calculate the *MAPE* value predicted by the exponential smoothing model, and this can be found below in Table 4.

The KNN1 is the model with a fixed T but improved K value. One day is divided into 48 time periods, so KNN1 model set the state vector T = 47. According to the minimum *MAPE*, the optimal K value is calculated, and the prediction results are shown in Table 4.

The LSTM model uses the MinMaxScaler scaler; all data are scaled between [0,1] to speed up convergence. The data in the form of time series is transformed into the form of supervised learning set, that is, the former number is taken as the input and the latter number as the corresponding output. An LSTM model was constructed and trained. The number of samples was 1, the number of trainings was 3, and the number of neurons in the LSTM layer was 5. After the predicted value is obtained, inverse scaling and inverse differentiation are performed to restore it to the original value range and traverse all test set data. The above operations are performed on each row of data and the final predicted value is saved; the prediction results are shown in Table 4.

**Figure 7:**
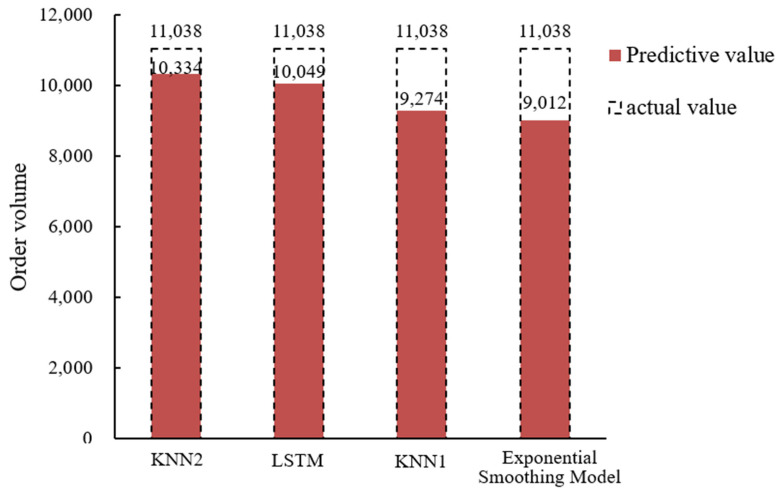
Comparison of the predicted values of online car-hailing demand by different models.

The KNN2 model is based on the above method to determine the appropriate T value and K value. For the time unit of 14:30, the corresponding optimal T value is 18 and K value is 9; Finally, the predicted value and *MAPE* value are filled in Table 4 below. The comparison of the prediction results of the four methods is shown in Figure 7.

From Figure 6 and Table 4, it can be concluded that exponential smoothing model is modified on the basis of simple historical average model, but it lacks the ability to identify the turning point of the data, and the prediction accuracy is still low compared with KNN model. The KNN1 model optimizes the K value, but does not consider the influence of the fixed T value on the prediction results; compared with the exponential smoothing model, the prediction accuracy is improved while it is still low compared with the KNN2 model, indicating that the consideration of increasing the T value can make the prediction of the model more accurate. The LSTM model has an advantage in the time series problem because of its internal forgetting layer structure, which is relatively suitable for solving the problem of online car-hailing demand forecasting; the prediction results are relatively smooth and the average absolute percentage error can be optimized to 8.96%, which is similar to the prediction accuracy of the KNN2 model proposed in this paper. We believe that if the relevant parameters of LSTM are further optimized, its prediction effect can be further improved. The KNN2 model can better adapt to the prediction of the fluctuation data of the online car-hailing. Finally, the average absolute percentage errors of the exponential smoothing model, KNN1 model, LSTM model and the KNN2 model are 18.35%, 15.98%, 8.96% and 6.38%. Compared with the other three prediction methods, the prediction accuracy of the KNN2 model can reach 93.62%, which is much higher than the exponential smoothing model (81.65%) and the KNN1 model (84.02%), similar to LSTM model (91.04%). Yu Bin stated that the K-nearest neighbor prediction model has a high prediction accuracy in short-term predictions [22], and the research in this paper also verifies this point of view. It can be seen that the K-nearest neighbor prediction model has high prediction accuracy and applicability in the short-term prediction of online car-hailing orders.

In summary, when comparing the four prediction methods through the analyses in this paper, it can be ascertained that the K-nearest neighbor algorithm is simple in theory, easy to implement, has a high accuracy, the highest prediction accuracy and a stronger adaptive ability. It can change with predicted environmental conditions, and through the classification of historical data sets and the adjustment of search algorithms and related parameters, the appropriate T value and K value are adopted. It can more accurately predict the demand for online car-hailing in cities, has good applicability in real time, and is more suitable for the short-term prediction of complex mutations, as well as being able to predict the trend of data changes in real time.

## 5. Conclusions

This paper takes the short-term forecasting of urban online car-hailing demand in Hefei as the research object and standardizes its historical order data from 2019 to 2021. The data set is divided into 48 categories within a day as the data-set unit. With the goal of the short-term prediction of the order demand of the urban online car-hailing platform, an adaptive K-nearest neighbor model prediction model is constructed. Aiming to minimize the average absolute percentage error, the values of the state vector T value and the number of neighbors K value are optimized, which can effectively prevents prediction inaccuracy.

An example data analysis shows that the K-nearest neighbor prediction method has high accuracy in the field of the short-term prediction of online car-hailing demand. The accuracy rises as high as 93.62%, and the stability is very good. It can better adapt to the urban online car-hailing order data with large time fluctuations and unevenness.

## 6. Practical Implications and Directions for Further Research

The prediction model of the K-nearest neighbor algorithm has been verified using the data from Hefei City, but whether it can be adapted to other different types of cities requires further research. This paper has studied Hefei City as a whole, and in the process of online car-hailing operations and scheduling, data prediction in a smaller range may be required, for example, in a short-term forecast of the demand for online car-hailing in a certain transportation hub. It would be necessary to further mine the data in a smaller geographic space in order to better guide the practicality and applicability of the K-nearest neighbor algorithm model. In addition, this paper lacks the consideration of spatial information on the accuracy of prediction. If the space–time information conditions are available, the graph convolution network will be considered to predict the online car-hailing order volume. At the same time, we also hope that Transformer models are applied to the prediction of online taxi order volume in the future.

## Figures and Tables

**Table 1:** Raw Data.

Data	Time	Order Volume
2019-08-01	00:00:00	983
2019-08-01	00:30:00	775
……	……	……
2021-02-04	11:30:00	336
2021-02-04	12:00:00	258

**Table 2:** Online Car-hailing Order Data After Processing.

Data	1	2	3	4	5	6	7	8	……	48
2019/8/1	983	775	580	407	336	258	174	157	……	1213
2019/8/2	1243	766	537	365	311	218	176	136	……	1641
……	……	……	……	……	……	……	……	……	……	……
2021/1/30	1178	843	614	451	395	285	252	230	……	1879
2021/1/31	3466	2340	1826	1352	1239	825	709	622	……	3814

**Table 3:** K-nearest Neighbor Algorithm Prediction Results.

State Vector t Value	48 Periods	Sample Size	Optimal K Value	Mape
1	1	265	optimalK:25	6.478301769
1	2	265	optimalK:14	5.579560974
1	3	265	optimalK:12	5.930144421
1	4	265	optimalK:13	6.021969718
1	5	265	optimalK:6	6.386384177
1	6	265	optimalK:11	6.710132134
……	……	……	……	……
47	48	265	optimalK:6	17.97111374

**Table 4:** Comparison table of prediction errors of online car-hailing demand by different models.

Time	MAPE			
	Exponential Smoothing Model	KNN1 Model	LSTM Model	KNN2 Model
31 January 2021 14:30	18.35%	15.98%	8.96%	6.38%

## Data Availability

Not applicable.

## References

[1] Ye X., Ye Q., Yan X., Wang T., Chen J., Li S. (2021). Demand Forecasting of Online Car-Hailing with Combining LSTM + Attention Approaches. Electronics.

[2] Lu X., Guo Y. (2020). Short-term forecasting model of online car-hailing demand based on GWO-LSTM. Autom. Instrum..

[3] Wang J., Wu Q., Mao F., Ren Y., Chen Z., Gao Y. (2021). Influencing Factor Analysis and Demand Forecasting of Intercity Online Car-Hailing Travel. Sustainability.

[4] Xu L., Guo Y. (2020). Short-term prediction model of online car-hailing demand based on GWO-LSTM. Autom. Instrum..

[5] Lu X., Ma C., Qiao Y. (2021). Short-term demand forecasting for online car-hailing using ConvLSTM networks. Phys. A Stat. Mech. Its Appl..

[6] Ma G. (2020). Travel Behavior Analysis and Demand Forecast of Car-Hailing Users.

[7] Ahmed M.S., Cook A.R. (1979). Analysis of freeway traffic time-series data by using Box-Jenkinstechniques. Transp. Res. Rec..

[8] Wang S., Shen Z., Gong X. (2009). Determination and comparison standard of urban taxi demand. Spec. Econ. Zone.

[9] Ma X., Tao Z., Wang Y., Yu H., Wang Y. (2015). Long short-term memory neural network for traffic speed prediction using remote microwave sensor data. Transp. Res. Part C Emerg. Technol..

[10] Tian Y., Zhang K., Li J., Lin X., Yang B. (2018). LSTM-based Traffic Flow Prediction with Missing Data. Neurocomputing.

[11] Niu K., Wang C., Zhou X., Zhou T. (2019). Predicting Ride-Hailing Service Demand via RPA-LSTM. IEEE Trans. Veh. Technol..

[12] Vaswani A., Shazeer N., Parmar N., Uszkoreit J., Jones L., Gomez A.N., Kaiser Ł., Polosukhin I. Attention Is All You Need. Proceedings of the Advances in Neural Information Processing Systems.

[13] Li G., Zhong S., Xiang L., Chan S.H.G., Li R., Hung C.C., Peng W.C. (2021). A Lightweight and Accurate Spatial-Temporal Transformer for Traffic Forecasting. arXiv.

[14] Bi S., Yuan C., Liu S., Wang L., Zhang L. (2022). Spatiotemporal Prediction of Urban Online Car-Hailing Travel Demand Based on Transformer Network. Sustainability.

[15] Smith B.L., Demetsky M.J. (1997). Traffic flow forecasting: Comparison of modeling approaches. J. Transp. Eng..

[16] Liu Z., Du W., Yan D. (2017). Short-term traffic flow prediction based on the combination of K-nearest neighbor algorithm and support vector regression. Highw. Traffic Sci. Technol..

[17] Lin P., Chen L., Lei Y. (2018). Short-term prediction of subway passenger flow based on K-nearest neighbor pattern matching. J. South China Univ. Technol. (Nat. Sci. Ed.).

[18] Wang X., Chen X., Yang X. (2015). Prediction of Expressway Short-term Travel Time Based on K-Nearest Neighbor Algorithm. Chin. J. Highw..

[19] Zhang T., Chen X., Xie M. (2010). Short-term traffic flow prediction method based on K-nearest neighbor nonparametric regression. Syst. Eng. Theory Pract..

[20] Wang X., Ding W. (2019). A Short-term Traffic Prediction Method for Expressway Big Data. Comput. Appl..

[21] Zhou X., Feng Q., Sun L. (2006). Short-term traffic flow prediction based on nearest neighbor method. J. Tongji Univ. (Nat. Sci. Ed.).

[22] Yu B., Wu S., Wang M. (2012). K-nearest short-term traffic flow prediction model. Transp. Eng..

[23] Zhu B. (2019). Application of Improved K-Nearest Neighbor Algorithm in Passenger Flow Prediction of Urban Rail Transit.

[24] Lei S. (2017). Prediction of Urban Road Short-Term Traffic Flow Based on Bayesian Classification and K-Nearest Neighbor Method.

[25] Cover T.M., Hart P.E. (1967). Nearest neighbour pattern classification. IEEE Trans. Inf. Theory.

[26] Yang T. (2019). Research on the Supervision of Online Car-Hailing in Hefei.

[27] Chen J. (2017). Short-Term Traffic Flow Prediction Based on K-Nearest Neighbor Nonparametric Regression Method.

